# Disposable Multi-Walled Carbon Nanotubes-Based Plasticizer-Free Solid-Contact Pb^2+^-Selective Electrodes with a Sub-PPB Detection Limit †

**DOI:** 10.3390/s19112550

**Published:** 2019-06-04

**Authors:** Yueling Liu, Yingying Gao, Rui Yan, Haobo Huang, Ping Wang

**Affiliations:** 1 Department of Food Quality and Safety, College of Management, Shanghai Sanda University, Shanghai 201209, China; 2 State Key Laboratory of Bioreactor Engineering, Biomedical Nanotechnology Center, School of Biotechnology, East China University of Science and Technology, Shanghai 200237, China; yingyinggao_7@163.com (Y.G.); yanrui268@126.com (R.Y.); huanghaobo@outlook.com (H.H.)

**Keywords:** solid contact ion-selective electrode, plasticizer-free, methyl methacrylate-*n*-butyl acrylate, Multi-walled carbon nanotubes, Pb(II)

## Abstract

Potentiometric plasticizer-free solid-contact Pb^2+^-selective electrodes based on copolymer methyl methacrylate-*n*-butyl acrylate (MMA-BA) as membrane matrix and multi-walled carbon nanotubes (MWCNTs) as intermediate ion-to-electron transducing layer have been developed. The disposable electrodes were prepared by drop-casting the copolymer membrane onto a layer of MWCNTs, which deposited on golden disk electrodes. The obtained electrodes exhibited a sub-ppb level detection limit of 10^−10^ mol·L^−1^. The proposed electrodes demonstrated a Nernstian slope of 29.1 ± 0.5 mV/decade in the linear range from 2.0 × 10^−10^ to 1.5 × 10^−3^ mol·L^−1^. No interference from gases (O_2_ and CO_2_) or water films was observed. The electrochemical impedance spectroscopy of the fabricated electrodes was compared to that of plasticizer-free Pb^2+^-selective electrodes without MWCNTs as intermediated layers. The plasticizer-free MWCNTs-based Pb^2+^-selective electrodes can provide a promising platform for Pb(II) detection in environmental and clinical application.

## 1. Introduction

Potentiometry with ion-selective electrodes (ISEs) is attractive for practical applications in many fields, for example, medical diagnosis and environmental monitoring [1,2,3]. The detection limit for most traditional liquid-contact electrodes based on plasticized poly(vinyl chloride) membrane was limited to micromolar range mainly due to the primary ion leaching from the inner filling solutions [4,5]. Conventional Pb^2+^-selective liquid-contact electrodes were usually prepared by incorporating a disk of elastic membrane into Philips-type electrode bodies and adding primary ions in the inner filling solutions. One solution is the addition of complexing agent (e.g., EDTA or NTA) [6], interfering ions (Et_4_NNO_3_), [7] or ion-exchange resins (Dowex C−350) [8] to precisely control the primary ion activity in the inner filling solutions. For example, the Pb^2+^-selective liquid-contact electrodes with ethylenediaminetetraacetic acid disodium salt Na_2_ (EDTA)-buffered inner solution exhibited a picomolar detection limit [9]. This type of Pb^2+^-selective liquid-contact electrodes were made of membranes glued to PVC tubing and the inner filling solutions (10^−3^ mol·L^−1^ Pb(NO_3_)_2_ and 5 × 10^−2^ mol·L^−1^ Na_2_(EDTA)) in contact with the reference element (Ag/AgCl in 3 mol·L^−1^ KCl) through a 1 mol·L^−1^ KCl bridge electrolyte. After the electrodes were conditioned in 10^−3^ mol·L^−1^ Pb(NO_3_)_2_ for more than 2 days, measurements were performed with a Metrohm NET-Titrino 721 at 22–23 °C in successively diluted stock solutions of 10^−2^ mol·L^−1^ Pb(NO_3_)_2_ from higher to lower concentrations. However, the lifetime was less than a week. Other effort was focused on applying an external compensating current to the sensor element [10]. However, the current control method requires sophisticated instrumentation and complicated experimental procedure, since the compensation current is related to the membrane potential. A more attractive approach is the exclusion of the troublesome inner solutions from the sensor and incorporation of ion-to-electron conducting layers leading to the solid-contact ion-selective electrodes (SC-ISEs) [11,12]. The introduction of conducting polymers (CP) as ion-to-electron transducers experienced rapid growth owing to the advantages of the obtained sensor and outstanding performances, which were comparable to those of traditional liquid-contact electrodes in all aspects [13,14]. Some CP-based SC-ISEs have exhibited some problems with water layer formation and interference from gases or light [15,16,17]. The lower detection limit was much worse when the potentiometric water layer test was positive. Sutter et al. reported the presence of water films in electropolymerized poly(octylthiophene) (POT)-based SC-ISEs where a sub-Nernstian response with a detection limit of 10^−7.1^ mol·L^−1^ was observed [18]. POT was deposited on the Au disk electrodes by galvanostatic electropolymerization of 3-octylthiophene in a one-compartment three-electrode electrochemical cell. After POT coatings were rinsed and dried, a solution of Pb^2+^-selective membrane was drop-casted on POT-coated Au disk electrodes. Before determination by a custom-made 14-channel electrode monitor, the electrodes were conditioned for at least 2 days in 10^−5^ mol·L^−1^ and then overnight in 10^−9^ mol·L^−1^ Pb(NO_3_)_2_ (background: 10^−4^ mol·L^−1^ HNO_3_). When POT was deposited by solvent drop-casting method, the linear response of the potentiometric sensor was extended into the subnanomolar range (10^−9.3^ mol·L^−1^). Moreover, Michalska et al. found the discharge of electrochemically deposited poly(pyrrole) can damage the lower detection limit of CP-based SC-ISEs due to the analyte ion fluxes from the CP in the direction to the sample [10]. Even a minor ion flux can deteriorate the lower detection limit by the formation of an extensive diffusion layer at the membrane surface in the sample side. Although a compensating current was proposed, which also used in liquid−contact electrodes, the lower detection limit remained relatively high. For poly(pyrrole) and other CP with functional groups that can be protonated, the possible reason for compromising the lower detection limit is the accumulation of primary ions in conducting layers or in thin water films formed between the conducting layer and ion-sensing membrane during the conditioning procedure [10,19]. In addition, it is well established that the diffusion coefficient of the ion-sensing membrane is a key factor to the lower detection limit. Although a large number of Pb^2+^-selective electrodes reported are based on plasticized poly(vinyl chloride) membranes, reducing the diffusion coefficient of the sensing membrane can suppress the transmembrane ion flux, which is beneficial to the lower detection limit. For example, the utilization of self-plasticized membrane materials [20,21,22,23,24] or ionophore covalently attached to the membrane components [25,26,27,28] successfully reduced the diffusion coefficient and led to lower detection limit.

In general, various factors should be considered on improving the lower detection limit of the SC-ISEs, such as the ion-sensing membrane (reduced diffusion coefficients), ion-to-electron conducting layer [29,30], and conditioning procedure [31,32]. Recently, there has been a surge in the application of carbon nanomaterials, such as carbon nanotubes and bimodal pore C_60_ [33,34,35], as transducing layers for SC-ISEs owing to their high hydrophobicity, double layer capacitance, and absence in redox reaction. Multi-walled carbon nanotubes (MWCNTs) required the assistance of surfactants to obtain a well−dispersed suspension mixture, but our former work found the presence of surfactants could deteriorate the sensing performance [36]. Thus, the design of surfactant-free MWCNTs-based single-piece Pb^2+^-selective electrodes was demonstrated with a low detection limit of 4.0 × 10^−10^ mol·L^−1^. The single-piece Pb^2+^-selective membrane was prepared by introducing MWCNTs directly into 2-nitrophenyl octyl ether plasticized poly(vinyl chloride) membranes. After sonication, the suspension mixture was drop-casted on the golden disk electrodes evenly and the solvent was evaporated thoroughly at ambient temperature. Before measurements, they were conditioned in 10^−3^ mol·L^−1^ Pb^2+^ solution overnight and then 10^−9^ mol·L^−1^ Pb^2+^ solution for 2 days (background: 10^−4^ mol·L^−1^ HNO_3_). Then, potentials were recorded continuously with increasing Pb^2+^ concentrations from lower to higher concentrations by a 16-channel interface. 

In this work, MWCNTs-based Pb^2+^-selective SC-ISEs with a trace level analysis at sub-ppb concentrations are presented. The proposed potentiometric sensor is designed based on the following principles: (a) Reducing the diffusion coefficient by the employment of plasticizer-free copolymer methacrylate-*n*-butyl acrylate (MMA-BA), (b) the utilization of hydrophobic MWCNTs as a transducing layer deposited by solution-casting, (c) the dispersion of MWCNTs in plasticizer instead of surfactants to reduce the surfactants’ interference. To the best of our knowledge, this is the first report on MWCNTs-based Pb^2+^-selective SC-ISEs with copolymer MMA-BA as membrane matrix. The obtained sensor was investigated by electrochemical impedance spectroscopy (EIS). The possibility of the formation of a thin water film at the interface was probed with a potentiometric water layer test. The influences of gases on the potential stability were also studied. The results suggest that the proposed sensor is promising for environmental and clinical Pb(II) determination. 

## 2. Materials and Methods

### 2.1. Reagents

Methyl methacrylate (MMA), butyl acrylate (BA), 2-nitrophenyl octyl ether (NPOE), lead ionophore IV, and tetrahydrofuran (THF) were all purchased from Sigma-Aldrich (Switzerland). Sodium tetrakis[3,5-bis(trifluoromethyl) phenyl]borate (NaTFPB) was purchased from Dojindo Laboratories (Japan). MWCNTs (>97%) with 10–20 nm diameter and 5–15 μm length were obtained from Shenzhen Nanotech Port Company (Shenzhen, China) with no further purification and modification. Ethyl acetate, methylene chloride, 1,4-dioxane, and 2,2′-azobisisobutyronitrile (AIBN) were purchased in analytical reagent grade from Titan (Shanghai, China) and dried before use. Lead nitrate (Pb(NO_3_)_2_) and other salts were obtained from Sigma-Aldrich. Aqueous solutions were prepared by dissolving the appropriate salts in freshly deionized water (DI water, resistance 18.25 MΩ·cm, Millipore, Burlington, MA, USA).

### 2.2. Polymer Preparation and Characterization

**Figure 1:**
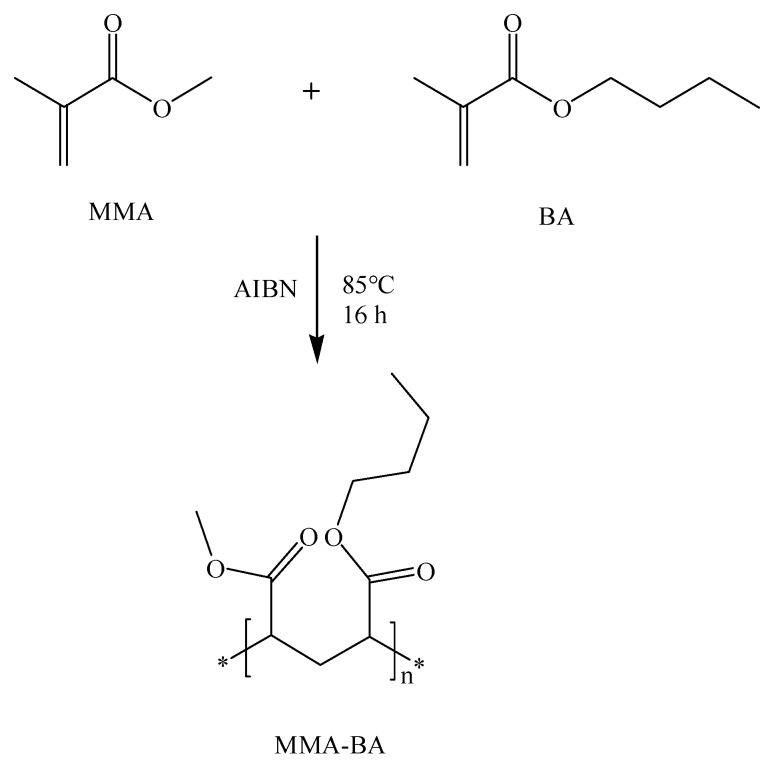
The free radical copolymerization reaction scheme of methyl methacrylate (MMA) and butyl acrylate (BA) resulting in plasticizer-free ion-selective membrane matrix.

**Figure 2:**
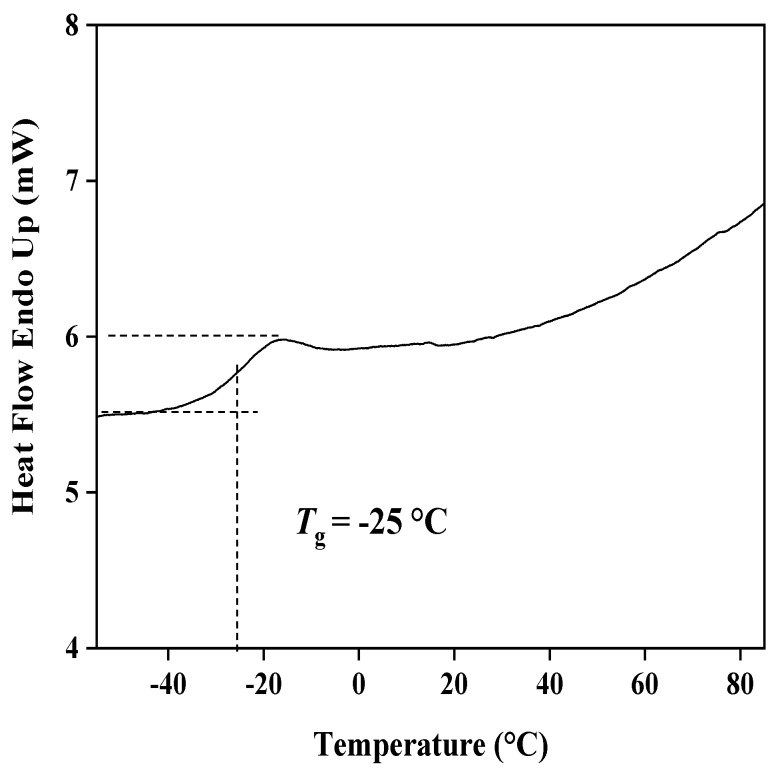
The glass-transition temperature (*T*_g_) of the copolymer MMA-BA.

The copolymers composed of MMA and BA were synthesized via thermally initiated free radical solution polymerization according to previous literature (see the scheme in Figure 1) [25]. Firstly, to remove the inhibitors, monomers MMA and BA were washed with a caustic solution (containing 5% (*w*/*v*) NaOH and 20% NaCl) in a 1:5 (monomer/caustic solution) ratio and water. The initiator AIBN was recrystallized from methanol and dried before use. Secondly, calculated amounts of monomers were added to 100 mL of dry ethyl acetate. The solution was degassed for 20 min by bubbling with nitrogen before the addition of 2,2′-azobisisobutyronitrile (AIBN). About 10 mg of AIBN was used for the polymerization. The homogeneous solution was continuously stirred and maintained at 85 °C for 16 h under an atmosphere of nitrogen. Thirdly, after the reaction was complete, the solvent was evaporated, and the precipitate was redissolved in 10 mL of 1,4-dioxane resulting in a gelatinous solution. Then, the gelatinous solution was added dropwise to 500 mL of DI water under vigorous stirring. The collected white precipitate was dissolved in 50 mL of methylene chloride, which was dried thoroughly and filtered by anhydrous Na_2_SO_4_. Finally, the transparent copolymer was obtained by evaporation and dried under vacuum at least for 2 days. The glass-transition temperature (*T*_g_) was detected by the differential scanning calorimeter (Diamond DSC, PerkinElmer, Waltham, MA, USA) and heated scanning at a rate of 10 °C/min. The *T*_g_ was then determined from DSC thermogram in Figure 2. The relative molecular mass of the copolymer was measured by gel permeation chromatography (GPC, Waters1515) with THF as solvent, as shown in Table 1.

### 2.3. Electrode Fabrication

Golden disk electrodes (Au, inner diameter (ID) = 2 mm, outside diameter (OD) = 6.35 mm) were applied for the fabrication of the proposed electrodes. They were polished with 0.3 μm alumina suspensions, rinsed with DI water, sonicated with ethanol and DI water separately, and finally dried under nitrogen. The cleaned electrodes were then tightly inserted into a piece of PVC tube (1 cm long, 5 mm ID and 8 mm OD) at the distal end.

The ion-selective sensing membrane cocktail (total mass 100 mg) was prepared by dissolving lead ionophore IV (2.0 wt %), NaTFPB (1.0 wt %), and MMA-BA (97 wt %) in 1 mL THF. The intermediate layer was prepared by dissolving 0.15 mg MWCNTs and 5 mg NPOE in 1 mL THF, and the mixture was sonicated for at least 20 min to obtain a uniform suspension, in a similar way to previous literature [36].

For MWCNTs-modified SC-ISEs, 100 μL of the MWCNTs suspension was drop-casted on the bare golden disk electrodes. The MWCNTs coatings were left to dry thoroughly in a desiccator. For MWCNTs-modified Pb^2+^˗selective SC-ISEs, 100 μL of the ion-selective sensing membrane cocktail was evenly drop-casted on the MWCNTs-modified SC-ISEs in a desiccator. After the solvent evaporation, the fabricated electrodes were conditioned in 10^−5^ mol·L^−1^ Pb^2+^ solution for 2 days and then 10^−10^ mol·L^−1^ Pb^2+^ solution for 1 day before measurements. All the Pb(NO_3_)_2_ solutions had the same background of 10^−4^ mol·L^−1^ HNO_3_ (pH = 3.8) in which Pb^2+^ is the predominating form of lead [37].

### 2.4. Apparatus and Measurements

The potentiometric responses were measured with a 16−channel interface (Lawson Labs, Inc.) controlled by a PCI−6281 data acquisition board and LabView 8.5 software (National Instruments, Austin, TX, USA). A double-junction Ag/AgCl/3 mol·L^−1^ KCl reference electrode containing 1 mol·L^−1^ CH_3_COOLi bridge electrolyte by Metrohm Ion Meter (Switzerland) was used. Different amounts of lead ions in the concentration range from 2.0 × 10^−12^ to 1.5 × 10^−3^ mol·L^−1^ were added progressively to 1.0 L of 10^−4^ mol·L^−1^ HNO_3_ solution in a crystallizing dish (200 mm). Before measurements, the crystallizing dish was washed with 10^−1^ mol·L^−1^ HNO_3_ solutions and pretreated overnight in 10^−4^ mol·L^−1^ HNO_3_ under magnetic stirring. The stability in time was measured by recording potentials of the developed electrodes consecutively under magnetic stirring. The activities of the ions were based on the activity coefficients, which were calculated according to the extended Debye−Hückel equation [38]. All the SC-ISEs’ potential results were the average of sets of at least three membranes, which were performed in laboratory ambient temperature.

The electrochemical impedance spectroscopy (EIS) measurements were performed in 1.5 × 10^−3^ mol·L^−1^ Pb(NO_3_)_2_ solution at room temperature, within the frequency range between 0.01 Hz to100 kHz using 100 mV amplitude at 0.2 V. All measurements were performed with a CHI 760D electrochemical workstation (Shanghai Chenhua Apparatus Corporation, Shanghai, China) with a Ag/AgCl/3 mol·L^−1^ KCl as reference electrode and a platinum as counter electrode.

## 3. Results and Discussion

### 3.1. Characterization of the Copolymer

This work reports the first plasticizer-free MWCNTs-based Pb^2+^-selective SC-ISEs based on the copolymer MMA-BA. Former literature pointed out that copolymer with *T*_g_ between −20 to −44 °C had the proper physical and mechanical property for the ion-sensing membranes and functionality of an ionophore when incorporated into the membranes [39,40]. Since a low *T*_g_ of the copolymer is critical to the functional polymeric ion-sensing membranes, the Fox equation was utilized to calculate the approximate *T*_g_ of the copolymer based on the weight fractions and *T*_g_ of the respective monomers (*T*_g (polyMMA)_ = 378 K, *T*_g (polyBA)_ = 218 K) [40]. In this study, to obtain a low *T*_g_ below −20 °C, the calculated weight fraction of MMA-BA is about 1:3. As can been seen from Figure 2, the fabricated MMA-BA shows a low experimental *T*_g_ of −25 °C. The resulting product also has a polydispersity of 1.57 and ${\bar{M}}_{w}$ of about 15,487 (Table 1), giving an elastic and tough film, which correlates well with former reports [25,40]. The results indicate that it may have the right characteristics to function as Pb^2+^-selective membrane without plasticizer. Since the native anionic sites in the membrane matrix can lead to a Nernstian response even in the absence of anionic additives NaTFPB [25], the potentiometric response of the membranes made of MMA-BA and lead ionophore IV was studied without NaTFPB. The blank copolymer membranes showed no response to ions, which indicates there is few ionic impurities. Then, the copolymer MMA-BA was applied as membrane matrix for the fabrication of MWCNTs-based Pb^2+^-selective SC-ISEs. Our previous work demonstrates that the existence of surfactants deteriorated the sensitivity of electrodes [36]. Thus, to avoid the potential interference from surfactants in the intermediate layer, MWCNTs were suspended in plasticizer NPOE in the aid of sonication. Subsequently, the obtained plasticizer-free MWCNTs-based Pb^2+^-selective SC-ISEs were characterized in terms of potentiometric response, impedance measurements, and so on.

### 3.2. Potentiometric Behavior

**Figure 3:**
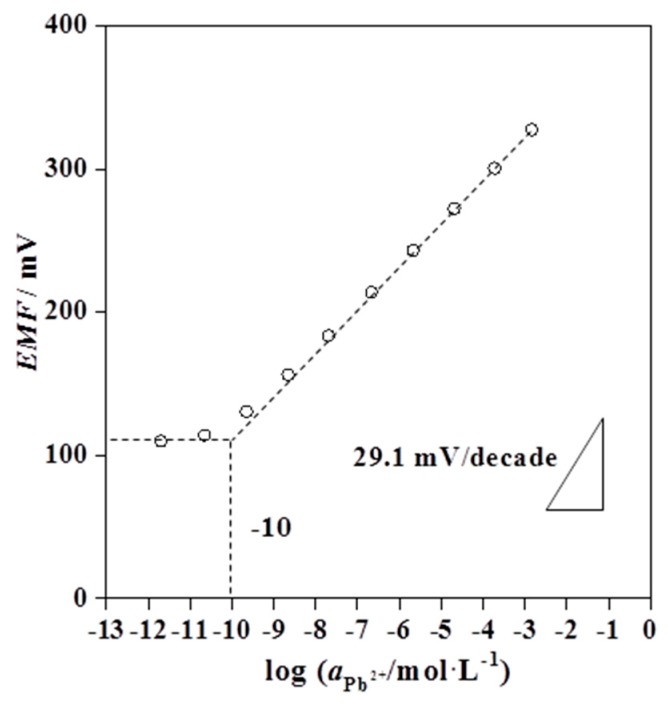
Potentiometric calibration curve of the multi-walled carbon nanotube (MWCNTs)-based plasticizer-free solid-contact Pb^2+^-selective electrodes in Pb^2+^ concentration range from 2.0 × 10^−12^ to 1.5 × 10^−3^ mol·L^−1^.

**Figure 4:**
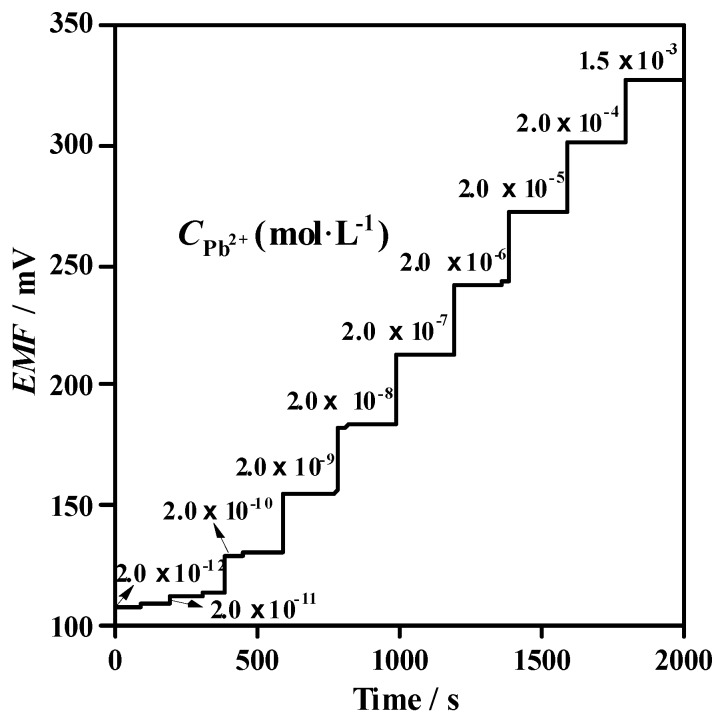
Dynamic potentiometric response of Au/MWCNTs/(MMA-BA)-Pb^2+^-ISEs with increasing Pb^2+^ concentrations from 2.0 × 10^−12^ to 1.5 × 10^−3^ mol·L^−1^.

The potentiometric response of the plasticizer-free MWCNTs-based Pb^2+^-selective SC-ISEs (Au/MWCNTs/(MMA-BA)-Pb^2+^-ISEs) was recorded in the Pb^2+^ concentration range from 2.0 × 10^−12^ to 1.5 × 10^−3^ mol·L^−1^. The proposed electrodes showed a Nernstian response of 29.1 ± 0.5 mV/decade over a linear range from 2.0 × 10^−10^ to 1.5 × 10^−3^ mol·L^−1^, as shown in Figure 3. A sub-ppb detection limit of 10^−10^ mol·L^−1^ is observed, which is calculated as the intersection of the two slopes (Figure 3). Table 2 displays the response characteristics and sensor construction of Au/MWCNTs/(MMA-BA)-Pb^2+^-ISEs in comparison with those of reported Pb^2+^-selective SC-ISEs with lead ionophore IV. As can been seen from Table 2, the proposed Au/MWCNTs/(MMA-BA)-Pb^2+^-ISEs show the lowest detection limit so far, down to 0.1 ppb for Pb^2+^ among available Pb^2+^-selective membrane with lead ionophore IV. Additionally, as shown in Figure 4, the developed electrodes exhibit fast response time of less than 30 s with a drift below 4 μV/s, which is much smaller than that of Au/POT/(MMA-DMA)-Pb^2+^-ISEs (0.4 mV/min) [18].

**Figure 5:**
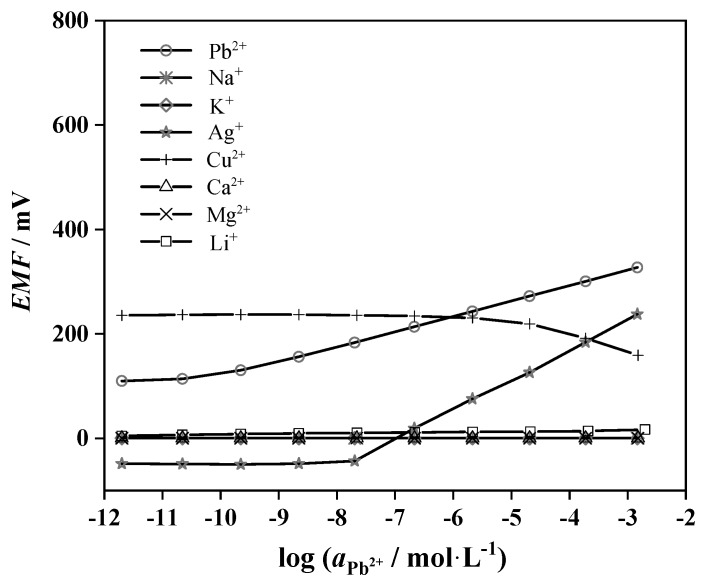
The response slopes profile for Pb^2+^ (○) and interfering ions, including Na^+^ (∗), K^+^ (◇), Ag^+^ (☆), Cu^2+^ (+), Ca^2+^ (△), Mg^2+^ (×), and Li^+^ (□).

Figure 5 demonstrates the response slopes profile for different interfering ions in selectivity determination. It is clear that the sensitivity of the developed electrodes on Pb^2+^ (○) is much higher than those of interfering ions, including Na^+^ (∗), K^+^ (◇), Ca^2+^ (△), Mg^2+^ (×), and Li^+^ (□). The proposed electrodes show a near-Nernstian response of 57.1 ± 0.8 mV/decade over the Ag^+^ concentration range from 2.0 × 10^−8^ to 2.0 × 10^−3^ mol·L^−1^. With increasing concentrations from 2.0 × 10^−^^5^ to 2.0 × 10^−3^ mol·L^−1^, the existence of Ag^+^ (☆) would cause a slightly minor interference on the sensitivity for Pb^2+^. However, obvious interference is found from Cu^2+^ (+) below the Pb^2+^ concentration of 2.0 × 10^−6^ mol·L^−1^. In other words, at concentrations lower than 2.0 × 10^−6^ mol·L^−1^, the sensitivity of the developed electrodes on Cu^2+^ is higher than that of Pb^2+^, so the electrodes will not work if both Cu^2+^ and Pb^2+^ are present at concentrations below 2.0 × 10^−6^ mol·L^−1^. Such interference from Ag^+^ or Cu^2+^ is often observed in Pb^2+^-selective electrodes [46,47]. The selectivity coefficients of the plasticizer-free MWCNTs-based Pb^2+^-selective SC-ISEs were evaluated using the International Union of Pure and Applied Chemistry (IUPAC) separate solution method (SSM, calculated at the highest ion concentration tested) [48]. As shown in Table 3, potentiometric selectivity coefficients of the proposed electrodes are comparable to those of reported Pb^2+^-selective SC−ISEs, such as Au/PPy/(PVC-DOS)-Pb^2+^-ISEs [19], Au/POT/(MMA-DMA)-Pb^2+^-ISEs [18] and Au/MEH-PPV/(PVC-NPOE)-Pb^2+^-ISEs [43].

### 3.3. Impedance Measurements

**Figure 6:**
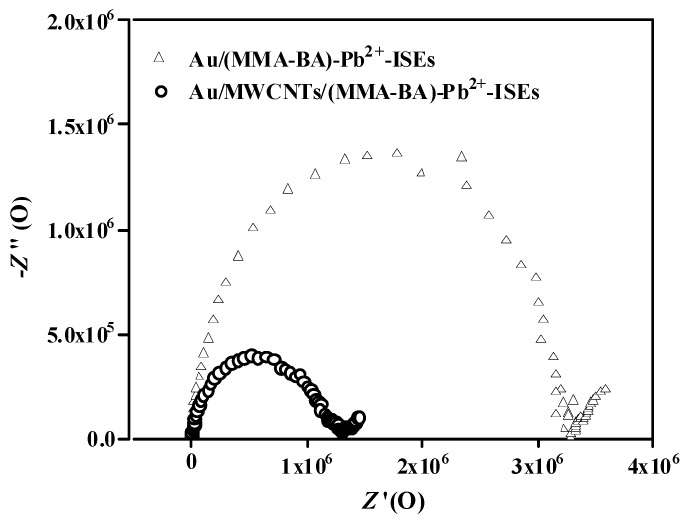
Impedance spectra of Au/(MMA-BA)-Pb^2+^-ISEs (triangle) and Au/MWCNTs/(MMA-BA)-Pb^2+^-ISEs (circle) in 1.5 × 10^−3^ mol·L^−1^ Pb^2+^ solution. *E*_dc_, 0.2 V; excitation amplitude, 100 mV; frequency range, 0.01 Hz−100 kHz.

Impedance measurements were performed to evaluate the electrochemical properties of the proposed electrodes. Figure 6 compares the EIS spectra of the plasticizer-free MWCNTs-based Pb^2+^-selective SC-ISEs (Au/MWCNTs/(MMA-BA)-Pb^2+^-ISEs, circle) and plasticizer-free Pb^2+^-selective electrodes in the absence of the MWCNTs layer (Au/(MMA-BA)-Pb^2+^-ISEs, triangle). The Au/(MMA-BA)-Pb^2+^-ISEs (triangle) exhibit a large semicircle in the high-frequency region, which arises from the bulk resistance and geometric capacitance of the ISM. The bulk resistances, which are estimated by the diameter of the high−frequency semicircle, are 3.28 and 1.29 MΩ for Au/(MMA-BA)-Pb^2+^-ISEs (triangle) and Au/MWCNTs/(MMA-BA)-Pb^2+^-ISEs (circle), respectively. The lower bulk resistance value of Au/MWCNTs/(MMA-BA)-Pb^2+^-ISEs (circle) suggests that the charge transport across the interface is facilitated greatly due to the presence of the MWCNTs as conducting layer. In addition, the low-frequency region of the Au/(MMA-BA)-Pb^2+^-ISEs (triangle) can be attributed to the charge-transfer resistance in parallel with a double layer capacitance at the interface between the membrane and Au substrate. The negligible low-frequency part in the EIS spectrum of Au/MWCNTs/(MMA-BA)-Pb^2+^-ISEs (circle) illustrates the higher double layer capacitance compared to that of Au/(MMA-BA)-Pb^2+^-ISEs (triangle). These results indicate that the introduction of the MWCNTs layer facilitates the charge transfer and ion-to-electron transduction effectively in plasticizer-free Pb^2+^-selective electrodes based on copolymer MMA-BA matrix.

### 3.4. Influence of Oxygen and Carbon Dioxide

**Figure 7:**
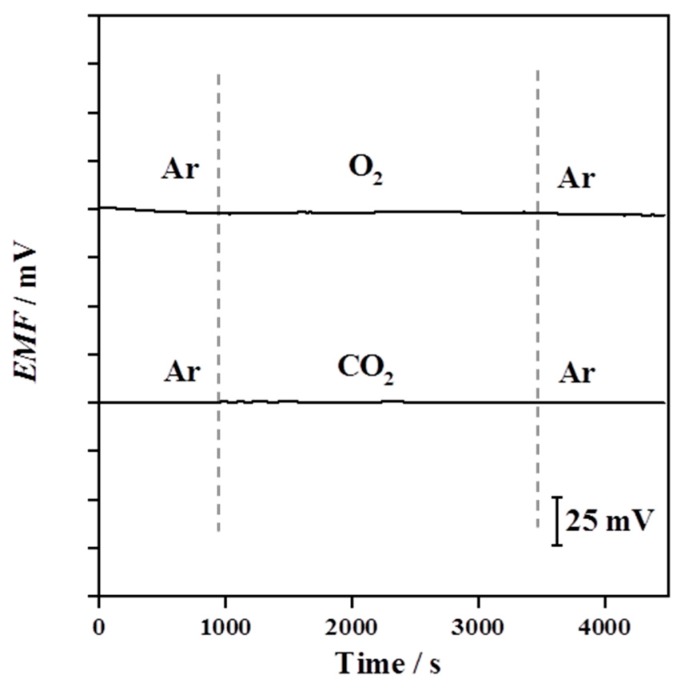
Influence of O_2_ (top) and CO_2_ (bottom) on the potential stability of Au/MWCNTs/(MMA-BA)-Pb^2+^-ISEs, which were immersed in a 1.5 × 10^−3^ mol·L^−1^ Pb(NO_3_)_2_ solution. For clarity, the potential responses of theses electrodes have been shifted vertically.

The importance of a MWCNTs solid-contact layer is demonstrated by Figure 7. Interferences from O_2_ and CO_2_ have been reported from several SC-ISEs where gases can easily permeate through the polymeric membrane and cause disturbances at the surface of the Au substrate [15,16]. More specifically, O_2_ can form an oxygen half-cell affecting the phase boundary potential, while CO_2_ can change the local pH at the electrode surface [49]. Therefore, the effects of O_2_ and CO_2_ on the potential stability of the Au/MWCNTs/(MMA-BA)-Pb^2+^-ISEs were investigated. The gas concentrations (O_2_ or CO_2_) were adjusted by bubbling these gases or Ar through the Pb(NO_3_)_2_ solutions (1.5 × 10^−3^ mol·L^−1^). As exhibited in Figure 7, the Au/MWCNTs/(MMA-BA)-Pb^2+^-ISEs display good potential stability when exposed to O_2_ or CO_2_. The outcome suggests that gases barely reach into the surface of the metal contact, which is probably due to the hydrophobicity of MWCNTs [36].

### 3.5. Potentiometric Water Layer Test

**Figure 8:**
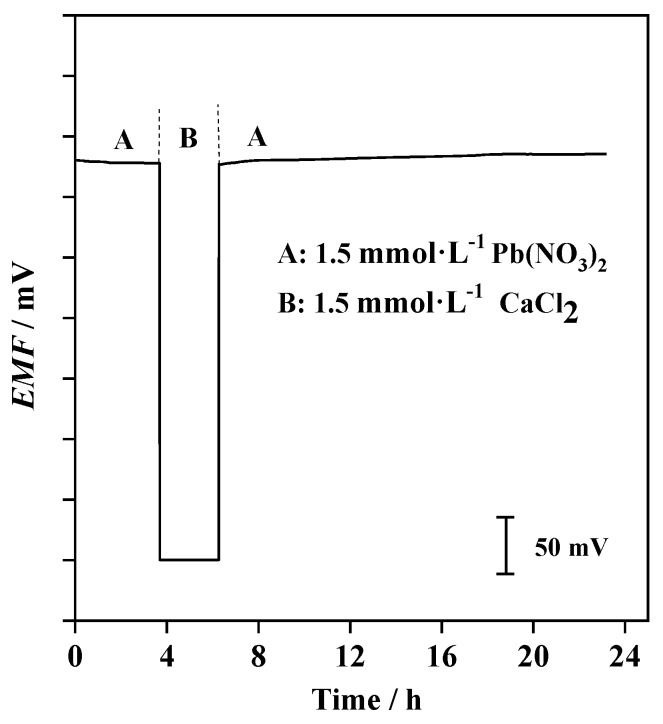
Potential water layer test of Au/MWCNTs/(MMA-BA)-Pb^2+^-ISEs; the measurements were switched between 1.5 mmol·L^−1^ Pb(NO_3_)_2_ and 1.5 mmol·L^−1^ CaCl_2_.

The potential water film at the ion-sensing membrane/electron conductor interface acts as a localized microscopic water pool in which primary ions may accumulate [50]. The leaching of primary ions into the sample during measurements can result in poor lower detection limit. Thus, potentiometric water layer test was carried out for the plasticizer-free MWCNTs-based Pb^2+^-selective SC-ISEs. As indicated in Figure 8, the proposed electrodes were firstly conditioned in the primary ion solution of 1.5 mmol·L^−1^ Pb(NO_3_)_2_. A stable potential for about 3.7 h was initially observed in Figure 8. After the primary ion solution was replaced with a discriminated interfering ion solution of 1.5 mmol·L^−1^ CaCl_2_, the immediate large potential shift was recorded. This phase boundary potential change corresponds well to the high selectivity behavior of plasticizer-free MWCNTs-based Pb^2+^-selective SC-ISEs (Table 3). After the CaCl_2_ solution was successively changed by the initial primary ion solutions, the stable potential response for nearly 17 h revealed the elimination of the undesirable water layer.

## 4. Conclusions

This work demonstrates for the first time that a 0.1 ppb limit of detection for lead(II) was achieved by the disposable plasticizer-free Pb^2+^-selective SC-ISEs based on the copolymer MMA-BA as membrane matrix and MWCNTs as a conducting layer. With good physical and mechanical properties, the copolymer MMA-BA is suitable for the fabrication of plasticizer-free Pb^2+^-selective SC-ISEs. The obtained electrodes show a Nernstian response of 29.1 ± 0.5 mV/decade within the concentration range from 2.0 × 10^−10^ to 1.5 × 10^−3^ mol·L^−1^ Pb^2+^ solution. Additionally, with high bulk capacitance and double layer capacitance, the proposed electrodes showed great potential stability due to the introduction of the MWCNTs layer. Moreover, the plasticizer-free MWCNTs-based Pb^2+^-selective SC-ISEs exhibited no obvious potential drift when exposed to O_2_ and CO_2_. The potentiometric water layer test confirms the absence of water films between the ion-selective membrane and the inner electron conductor. This work indicates that potentiometric solid-contact ion-selective electrodes for lead(II) detection has reached a performance well comparable to most advance methods.

## Figures and Tables

**Table 1:** The physical properties of the copolymer MMA-BA.

Composition Based on Feed (mol Fraction)	Average Molecular Weight (Daltons)						Polydispersity
	${\bar{M}}_{n}$	${\bar{M}}_{w}$	$\bar{MP}$	${\bar{M}}_{z}+1$	${\bar{M}}_{z}/{\bar{M}}_{w}$	${\bar{(M}}_{z}+1)/{\bar{M}}_{w}$	
MMA/BA = 1:3	9837	15,487	12,441	31,759	1.50	2.05	1.57

**Table 2:** Response characteristics and sensor construction of Au/MWCNTs/(MMA-BA)-Pb^2+^ ion-selective electrodes (ISEs) in comparison with those of reported Pb^2+^-selective solid-contact electrodes.

Low Detection Limit (mol·L^−1^)	Response Slope (mV·decade^−1^)	Electrode Substrate	Ion-to-Electron Conducting Layer and Deposition Method	Ion-Selective Membrane Composition	Reference
1.0 × 10^−10^	29.1 ± 0.5	Au disk electrodes	multi-walled carbon nanotubes (MWCNTs)/solution drop-casting	PVC, NPOE, lead ionophore IV, NaTFPB	this work
6.3 × 10^−10^	29.1 ± 0.7	glassy carbon disk electrodes	electrospun polyaniline microfibers film/solution drop−casting	PVC, NPOE, lead ionophore IV, NaTFPB	[41]
5.0 × 10^−10^	28.8 ± 1.2	glassy carbon disk electrodes	bimodal pore C_60_/electrodeposition	PVC, NPOE, lead ionophore IV, ETH 500, NaTFPB	[34]
7.9 × 10^−10^	28.4 ± 0.4	glassy carbon disk electrodes	poly(3,4-ethylenedioxythiophene) doped with polystyrene sulfonate anion (PEDOT-PSS)/electrodeposition	PVC, NPOE, lead ionophore IV, potassium tetrakis(*p*-chlorophenyl)-borate	[42]
1.2 × 10^−9^	23.4 ± 0.0	screen printer electrodes	poly(3-octylthiophene-2,5-diyl)/solution drop−casting	PVC, NPOE, lead ionophore IV, NaTFPB	[32]
6.3 × 10^−10^	29.1 ± 0.8	Au disk electrodes	poly(2-methoxy-5-(2′-ethylhexyloxy)-*p*-phenylene vinylene) (MEH-PPV)/solution drop-casting	PVC, NPOE, lead ionophore IV, NaTFPB	[43]
1.0 × 10^−9^	26.2 ± 0.3	glassy carbon disk electrodes	poly(octylthiophene) (POT)/solution drop-casting	hydroxyethyl methacrylate-butyl acrylate, lead ionophore IV, NaTFPB	[44]
1.2 × 10^−8^	27.9 ± 0.3	glassy carbon disk electrodes	poly(3,4-dioctyloxythiophene) doped with lead ionophore IV/electrodeposition	PVC, DOS, lead ionophore IV, NaTFPB	[45]
5.0 × 10^−10^	Nernstian response	Au disk electrodes	poly(octylthiophene) (POT)/solution drop-casting	Methylmethacrylate-decylmethacrylate, ETH500, lead ionophore IV, NaTFPB	[18]

**Table 3:** The potentiometric selectivity coefficients ($\log K_{{\mathrm{Pb}}^{2+},J}^{\mathrm{pot}}$) of the proposed Au/MWCNTs/(MMA-BA)-Pb^2+^-ISEs compared to those available Pb^2+^-selective solid-contact electrodes with Au disk electrodes. (*n* = 3).

		Ion J $\left(\log K_{{\mathrm{Pb}}^{2+},J}^{\mathrm{pot}}\right)$						
	J	Na^+^	K^+^	Li^+^	Ca^2+^	Mg^2+^	Cu^2+^	Ag^+^
Electrode Type								
Au/MWCNTs/(MMA-BA)-Pb^2+^-ISEs		−8.49 ± 0.2	−8.48 ± 0.1	−7.93 ± 0.2	−11.07 ± 0.3	−11.07 ± 0.2	−5.70 ± 0.3	−0.47 ± 2.1
Au/PPy/(PVC-DOS)-Pb^2+^-ISEs [19]		−6.3 ± 0.1	−6.6 ± 0.1	Not given	−13.6 ± 0.2	Not given	Not given	Not given
Au/POT/(MMA-DMA)-Pb^2+^-ISEs [18]		−8.7 ± 0.2	−8.7 ± 0.2	Not given	−14.3 ± 0.2	Not given	Not given	Not given
Au/MEH-PPV/(PVC-NPOE)-Pb^2+^-ISEs [43]		−6.6 ± 0.1	Not given	Not given	−16.5 ± 0.2	Not given	−4.6 ± 0.1	Not given

## References

[1] Velde L., Angremont E., Olthuis W. (2016). Solid contact potassium selective electrodes for biomedical applications—A review. Talanta.

[2] Bakker E., Pretsch E. (2005). Potentiometric sensors for trace-level analysis. Trends Anal. Chem..

[3] Choosang J., Numnuam A., Thavarungkul P., Kanatharana P., Radu T., Ullah S., Radu A. (2018). Simultaneous detection of ammonium and nitrate in environmental samples using on ion-selective electrode and comparison with portable colorimetric assays. Sensors.

[4] Li X.G., Feng H., Huang M.R., Gu G.L., Moloney M.G. (2011). Ultrasensitive Pb (II) potentiometric sensor based on copolyaniline nanoparticles in a plasticizer-free membrane with a long lifetime. Anal. Chem..

[5] Golcs Á., Horváth V., Huszthy P., Tóth T. (2018). Fast potentiometric analysis of lead in aqueous medium under competitive conditions using an acridono-crown ether neutral ionophore. Sensors.

[6] Radu A., Peper S., Bakker E., Diamond D. (2007). Guidelines for improving the lower detection limit of ion-selective electrodes: A systematic approach. Electroanalysis.

[7] Szigeti Z., Malon A., Vigassy T., Csokai V., Grün A., Wygladacz K., Ye N., Xu C., Chebny V.J., Rathore R. (2006). Novel potentiometric and optical silver ion-selective sensors with subnanomolar detection limits. Anal. Chim. Acta.

[8] Radu A., Peper S., Gonczy C., Runde W., Diamond D. (2010). Trace-level determination of Cs^+^ using membrane-based ion-selective electrodes. Electroanalysis.

[9] Sokalski T., Ceresa A., Zwickl T., Pretsch E. (1997). Large improvement of the lower detection limit of ion-selective polymer membrane electrodes. J. Am. Chem. Soc..

[10] Michalska A., Maksymiuk K. (2004). Conducting polymer membranes for low activity potentiometric ion sensing. Talanta.

[11] Zdrachek E., Bakker E. (2019). Potentiometric Sensing. Anal. Chem..

[12] Bobacka J., Ivaska A., Lewenstam A. (2008). Potentiometric ion sensors. Chem. Rev..

[13] Bobacka J. (2010). Conducting polymer-based solid-state ion-selective electrodes. Electroanalysis.

[14] Lange U., Roznyatovskaya N.V., Mirsky V.M. (2008). Conducting polymers in chemical sensors and arrays. Anal. Chim. Acta.

[15] Vázquez M., Bobacka J., Ivaska A., Lewenstam A. (2002). Influence of oxygen and carbon dioxide on the electrochemical stability of poly (3,4-ethylenedioxythiophene) used as ion-to-electron transducer in all-solid-state ion-selective electrodes. Sens. Actuators B Chem..

[16] Mir M., Lugo R., Tahirbegi I.B., Samitier J. (2014). Miniaturizable ion-selective arrays based on highly stable polymer membranes for biomedical applications. Sensors.

[17] Yin T., Pan D., Qin W. (2012). A solid-contact Pb^2+^-selective polymeric membrane electrode with nafion-doped poly(pyrrole) as ion-to-electron transducer. J. Solid State Electrochem..

[18] Sutter J., Radu A., Peper S., Bakker E., Pretsch E. (2004). Solid-contact polymeric membrane electrodes with detection limits in the subnanomolar range. Anal. Chim. Acta.

[19] Sutter J., Lindner E., Gyurcsányi R.E., Pretsch E. (2004). A polypyrrole-based solid-contact Pb^2+^-selective PVC-membrane electrode with a nanomolar detection limit. Anal. Bioanal. Chem..

[20] Chumbimuni-Torres K.Y., Rubinova N., Radu A., Kubota L.T., Bakker E. (2006). Solid contact potentiometric sensors for trace level measurements. Anal. Chem..

[21] Lisak G., Grygolowicz-Pawlak E., Mazurkiewicz M., Malinowska E., Sokalski T., Bobacka J., Lewenstam A. (2009). A New polyacrylate-based lead (II) ion-selective electrodes. Microchim. Acta.

[22] Heng L.Y., Hall E.A. (2000). Producing “self-plasticizing” ion-selective membranes. Anal. Chem..

[23] Hernández R., Riu J., Bobacka J., Vallés C., Jiménez P., Benito A.M., Maser W.K., Rius F.X. (2012). Reduced graphene oxide films as solid transducers in potentiometric all-solid-state ion-selective electrodes. J. Phys. Chem. C.

[24] Parra E.J., Crespo G.A., Riu J., Ruiz A., Rius F.X. (2009). Ion-selective electrodes using multi-walled carbon nanotubes as ion-to-electron transducers for the detection of perchlorate. Analyst.

[25] Heng L.V., Chern L.H., Ahmad M. (2002). A hydrogen ion-selective sensor based on non-plasticised methacrylic-acrylic membranes. Sensors.

[26] Qin Y., Peper S., Bakker E. (2002). Plasticizer-free polymer membraneion-selective electrodes containing a methacrylic copolymer matrix. Electroanalysis.

[27] Liu Y., Xue Y., Tang H., Wang M., Qin Y. (2012). Click-immobilized K^+^-selective ionophore for potentiometric and optical sensors. Sens. Actuators B Chem..

[28] Püntener M., Vigassy T., Baier E., Ceresa A., Pretsch E. (2004). Improving the lower detection limit of potentiometric sensors by covalently binding the ionophore to a polymer backbone. Anal. Chim. Acta.

[29] Michalska A.J., Appaih-Kusi C., Heng L.Y., Walkiewicz S., Hall E.A. (2004). An experimental study of membrane materials and inner contacting layers for ion-selective K^+^ electrodes with a stable response and good dynamic range. Anal. Chem..

[30] Michalska A., Wojciechowski M., Bulska E., Maksymiuk K. (2010). Experimental study on stability of different solid contact arrangements of ion-selective electrodes. Talanta.

[31] Yu S., Yuan Q., Li F., Liu Y. (2012). Improved potentiometric response of all-solid-state Pb^2+^-selective electrode. Talanta.

[32] Anastasova S., Radu A., Matzeu G., Zuliani C., Mattinen U., Bobacka J., Diamond D. (2012). Disposable solid-contact ion-selective electrodes for environmental monitoring of lead with ppb limit-of-detection. Electrochim. Acta.

[33] Liang R., Yin T., Qin W. (2015). A simple approach for fabricating solid-contact ion-selective electrodes using nanomaterials as transducers. Anal. Chim. Acta.

[34] Li J., Yin T., Qin W. (2015). An all-solid-state polymeric membrane Pb^2+^-selective electrode with bimodal pore C_60_ as solid contact. Anal. Chim. Acta.

[35] Jaworska E., Lewandowski W., Mieczkowski J., Maksymiuk K., Michalska A. (2013). A Simple and disposable potentiometric sensors based on graphene or multi-walled carbon nanotubes–carbon–plastic potentiometric sensors. Analyst.

[36] Liu Y., Liu Y., Gao Y., Wang P. (2019). A general approach to one-step fabrication of single-piece nanocomposite membrane based Pb^2+^-selective electrodes. Sens. Actuators B Chem..

[37] Didina S.E., Mitnik L.L., Koshmina N.V., Grekovich A.L., Mikhelson K.N. (1994). Lead-selective electrodes based on liquid ion-exchangers. Sens. Actuators B Chem..

[38] Koryta J., Dvořák J. (1987). Principles of Electrochemistry.

[39] Qin Y., Peper S., Radu A., Ceresa A., Bakker E. (2003). Plasticizer-free polymer containing a covalently immobilized Ca^2+^-selective ionophore for potentiometric and optical sensors. Anal. Chem..

[40] Heng L.Y., Hall E.A. (2000). Methacrylic–acrylic polymers in ion-selective membranes: Achieving the right polymer recipe. Anal. Chim. Acta.

[41] Liu C., Jiang X., Zhao Y., Jiang W., Zhang Z., Yu L. (2017). A solid-contact Pb^2+^-selective electrode based on electrospun polyaniline microfibers film as ion-to-electron transducer. Electrochim. Acta.

[42] Guziński M., Lisak G., Sokalski T., Bobacka J., Ivaska A., Bocheńska M., Lewenstam A. (2013). Solid-contact ion-selective electrodes with highly selective thioamide derivatives of *p*-tert-butylcalix[4]arene for the determination of lead (II) in environmental samples. Anal. Chem..

[43] Yu S., Li F., Yin T., Liu Y., Pan D., Qin W. (2011). A solid-contact Pb^2+^-selective electrode using poly(2-methoxy-5-(2′-ethylhexyloxy)-*p*-phenylene vinylene) as ion-to-electron transducer. Anal. Chim. Acta.

[44] Michalska A., Wojciechowski M., Bulska E., Mieczkowski J., Maksymiuk K. (2009). Poly (*n*-butyl acrylate) based lead (II) selective electrodes. Talanta.

[45] Michalska A., Skompska M., Mieczkowski J., Zagórska M., Maksymiuk K. (2006). Tailoring solution cast poly (3,4-dioctyloxythiophene) transducers for potentiometric all-solid-state ion-selective electrodes. Electroanalysis.

[46] Li X., Ma X., Huang M. (2008). Lead(II) ion-selective electrode based on polyaminoanthraquinone particles with intrinsic conductivity. Talanta.

[47] Lee H.K., Song K., Seo H.R., Choi Y.K., Jeon S. (2004). Lead(II)-selective electrodes based on tetrakis(2-hydroxy-1-naphthyl)porphyrins: The effect of atropisomers. Sens. Actuators B Chem..

[48] Bakker E., Pretsch E., Bühlmann P. (2000). Selectivity of potentiometric ion sensors. Anal. Chem..

[49] Hu J., Zou X.U., Stein A., Bühlmann P. (2014). Ion-selective electrodes with colloid-imprinted mesoporous carbon as solid contact. Anal. Chem..

[50] Veder J., Marco R.D., Clarke G., Chester R., Nelson A., Prince K., Pretsch E., Bakker E. (2008). Elimination of undesirable water layers in solid-contact polymeric ion-selective electrodes. Anal. Chem..

